# Beyond bone mineral density, FRAX-based tailor-made intervention thresholds for therapeutic decision in subjects on glucocorticoid: A nationwide osteoporosis survey

**DOI:** 10.1097/MD.0000000000005959

**Published:** 2017-02-03

**Authors:** Shan-Fu Yu, Jia-Feng Chen, Yin-Chou Chen, Han-Ming Lai, Chi-Hua Ko, Wen-Chan Chiu, Fu-Mei Su, Chung-Yuan Hsu, Ben Yu-Jih Su, Chih-Hsing Wu, Tien-Tsai Cheng

**Affiliations:** a Division of Rheumatology, Allergy, and Immunology, Department of Internal Medicine, Kaohsiung Chang Gung Memorial Hospital; b Chang Gung University College of Medicine, Kaohsiung; c Department of Family Medicine, National Cheng Kung University Hospital, Tainan, Taiwan.

**Keywords:** bone mineral density, FRAX, glucocorticoid, intervention threshold, osteoporosis

## Abstract

Glucocorticoid-induced osteoporosis (GIOP) is the most common cause of secondary osteoporosis and confers a substantial risk for future fractures. Several recent guidelines for GIOP management have recommended the use of intervention thresholds to direct pharmacological therapy in those at high risk of fracture. The aim of this study was to analyze the characteristics of subjects on a glucocorticoid (GC) and to implement the Fracture Risk Assessment Tool (FRAX)-based intervention threshold for therapeutic decision-making.

This was a cohort substudy of a nationwide osteoporosis screening program conducted in Taiwan from 2008 to 2011. All participants were requested to complete a questionnaire including FRAX elements, and antiosteoporosis medication (AOM) history was assessed before bone mineral density (BMD) measurement. GC users were recruited as the study group. Controls comprised randomly selected age- and sex-matched non-GC users. Individual intervention threshold (IIT) was set at individual-specific FRAX probability of a major osteoporotic fracture, relative to subjects with prior fractures. The characteristics and calculated IIT of all participants were analyzed.

A total of 8704 participants were enrolled, including GC users (n = 807) and controls (n = 7897). There was no significant difference in BMD between GC users and controls. Clinical fracture risks, including previous fracture, parental hip fracture, rheumatoid arthritis, and secondary osteoporosis were higher in GC users than in controls. GC users had a higher 10-year probability of either major or hip fracture than controls. The proportion of GC users with a 10-year probability of major osteoporotic fracture above IIT was higher than in controls (75.0% vs 10.6%; *P* < 0.001). Only 20.3% of GC users and 30.5% of controls whose fracture risk was above IIT reported taking AOM.

These findings suggest that more GC users should receive active intervention based on IIT, regardless of BMD. However, less than one-fourth of GC users whose fracture risk was above IIT received AOM, indicating that GIOP is markedly undertreated. We recommend commencing AOM for GIOP according to IIT, instead of BMD alone.

## 1 Introduction

Glucocorticoids (GCs) are among the most frequently prescribed medications, and might be administered in up to 20% of all osteoporosis cases.^[1]^ Estimates suggest that 1.2% of the US population uses chronic GC therapy for various inflammatory and autoimmune disorders.^[2]^ An earlier study conducted in the UK estimated the frequency of oral GC use at approximately 0.5% of the general population, and 1.7% of women older than 55 years.^[3]^ Notably, GC therapy has not only been associated with bone loss, but also with a higher fracture risk.^[4,5]^ The fracture incidence is related to the daily dose and duration of GC exposure.^[4]^

Fracture occurs at a higher bone mineral density (BMD) in GC-treated individuals than in those not receiving GC,^[6,7]^ suggesting that fracture risk can only be partly attributed to BMD. It is therefore notable that conventional bone density testing is ineffective for detecting bone microarchitectural changes^[5]^ or predicting the severity of vertebral fractures in women undergoing GC therapy.^[8]^ Therefore, appropriate diagnostic procedures and physicians’ awareness of the fracture risks are of paramount importance to the identification and treatment of high-risk patients.^[9]^

The World Health Organization Fracture Risk Assessment Tool (FRAX) was established in 2008 to allow health providers to estimate individual 10-year probabilities of fragility-related fractures.^[10]^ The recent guidelines from the American College of Rheumatology (ACR) incorporated FRAX as an assessment tool for fracture risk of GC-induced osteoporosis (GIOP) in the 2010 revision.^[11]^ Based on new evidence regarding intervention thresholds in GIOP, the Joint GIO Guidelines Working Group of the International Osteoporosis Foundation and the European Calcified Tissue Society (IOF-ECTS) have published a framework for the development of guidelines for the management of GIOP in 2012.^[12]^ Taiwanese osteoporosis practice guidelines (Chinese version) proposed by the Taiwanese Osteoporosis Association (TOA) was updated in 2015. The recommendations by TOA for GIOP was based on the 2012 IOF-ECTS guideline, that patients should be categorized as previous fracture, menopausal status, GC dose, and age-dependent intervention threshold.

At present, few studies have investigated GIOP via the application of FRAX. The first purpose of this study was to analyze the characteristics of subjects on GC in a Taiwanese population by using FRAX. The secondary purpose of this study was to implement of FRAX-based intervention threshold in treatment decision.

## 2 Methods

### 2.1 Study population

The present study was based on the Taiwan OsteoPorosis Survey (TOPS) database, which is managed by the Taiwanese Osteoporosis Association.^[13]^ In brief, a BMD survey was conducted nationwide using a bus equipped with a dual-energy X-ray absorptiometry (DXA) scanner (Explorer; Hologic, Inc., Waltham, MA). Participants were recruited at 104 sites in Taiwan, including both urban and rural areas. BMD measurements included regions of the lumbar spine, total hip and femoral neck (FN). The inclusion criteria for analysis were a willingness to participate in the study and ability to read and provide informed consent. As this project was based on a screening program, no specific exclusion criteria were set. However, participants who could not access the machine installed in the bus or those with a previous history of bilateral hip fracture were excluded from the analysis.

### 2.2 Data collection and measurements

Each participant was interviewed by a well-trained research assistant to complete the structured questionnaire, which included the clinical risk factors specifically for FRAX calculation tool, before undergoing BMD measurement. The clinical risk factors comprised a prior history of fracture, a parental history of hip fracture, use of oral GC, rheumatoid arthritis (RA) and other secondary causes of osteoporosis, current smoking, and alcohol intake 3 or more units daily.

There is a current international consensus to use the Caucasian women aged 20 to 29 years from National Health and Nutrition Examination Survey III at FN as an “international” reference.^[14]^ The osteoporosis diagnosis for postmenopausal females and men age 50 and older is a *T* score of −2.5 or less at the FN.^[15,16]^ Premenopausal females and men younger than 50 with a *Z* score of −2.0 or lower should be defined as having a bone density that is “below the expected range for age.”^[17]^

**Figure 1:**
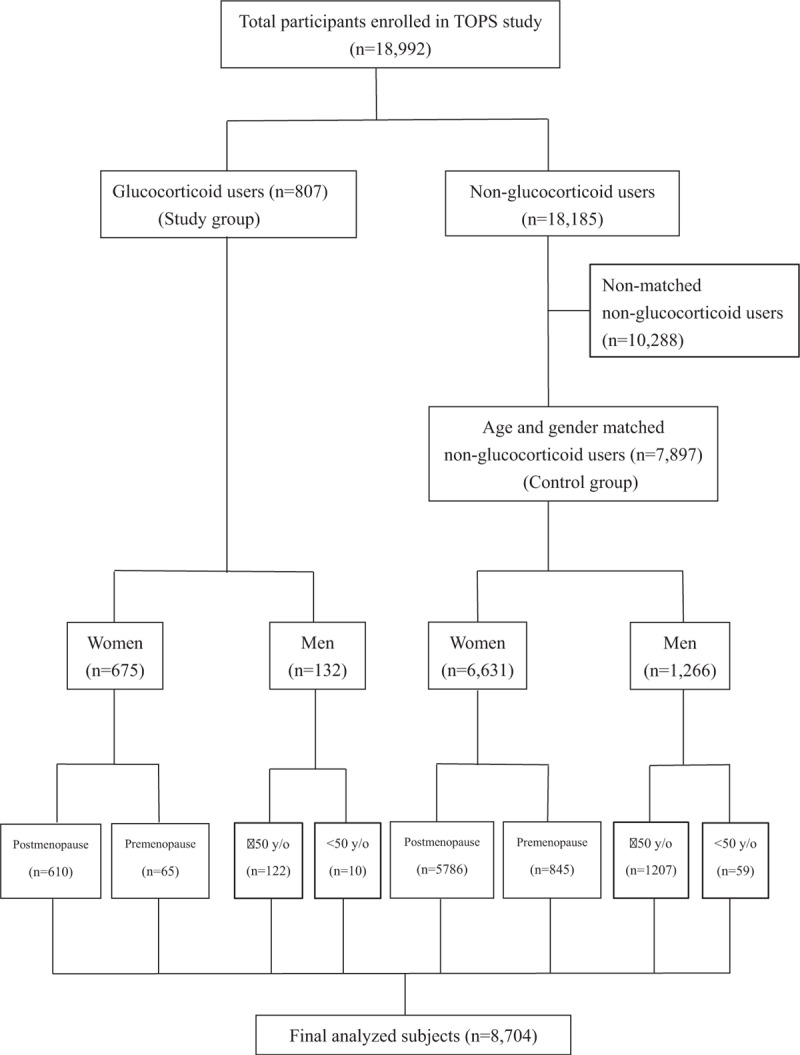
Characteristics of all participants.

Participants who were GC users, defined as those who answered “yes” to the questionnaire item “Have you currently taken GC for more than 3 months?” were selected as the study group. The control group comprised age- and gender-matched non-GC users randomly selected from among the screening program participants. Of the 18,185 eligible frequency age and gender-matched controls 7897 (43.4%) consented to participate. A study flowchart of patient disposition is shown in Fig. 1. We calculated the 10-year probabilities of major and hip fracture using the Taiwanese-specific FRAX. For major osteoporotic fracture, participants were categorized into low-risk, medium-risk, and high-risk groups based on the 10-year fracture risk probability calculated using FRAX with cut-off points of 10% and 20%.^[18]^

We followed the guidance by IOF/ECTS for GIOP to calculate the intervention threshold for each participant.^[12]^ We modified the calculation of individual intervention threshold (IIT) by input of gender, body weight, body height and assume that participant had a “prior fracture” but no other risk factors and BMD unavailable. Then, we recalculate the individual-specific 10-year probability of major fracture of that participant by input the real situation. We defined “above IIT” as 10-year probability of major fracture of that participant is higher or equal to IIT of the same participant. We compared the proportion of above IIT between the GC users and controls.

### 2.3 Ethics statement

This study was approved by the local Institutional Review Board of Chang Gung Memorial Hospital (102-1878B). All participants provided written informed consent to participate in this study.

### 2.4 Statistical analysis

All available cases were included rather than performing a formal sample size calculation to determine study size. The descriptive summary is presented in the form of mean ± standard deviation. Levene test was used to test the homogeneity of the sampling. Continuous variables were evaluated by paired *t* test or Wilcoxon signed rank test. Chi-square test, Fisher exact test, or conditional logistic regression was used for the qualitative variables. If a FRAX risk factor was missing what was recorded in the FRAX calculation, convention is generally to characterize the risk factor as not present. The software package PASW Statistics for Windows, Version 18.0 (SPSS, Inc., Chicago, IL) was used for the statistical analysis. A level of statistical significance of *P* < 0.05 was used for all statistical tests performed.

## 3 Results

A total of 18,992 participants, including 4323 (22.8%) males and 14,669 (77.2%) females, were enrolled in the TOPS program between 2008 and 2011 (Fig. 1). A total of 807 and 7897 participants were included in the GC user and control groups, respectively. The demographics of both groups are presented in Table 1. There were no differences in age, sex, age at menopause, body weight, body height, and body mass index between GC users and controls. The GC users had a higher frequency of menopause and premature menopause relative to controls.

**Table 1:**
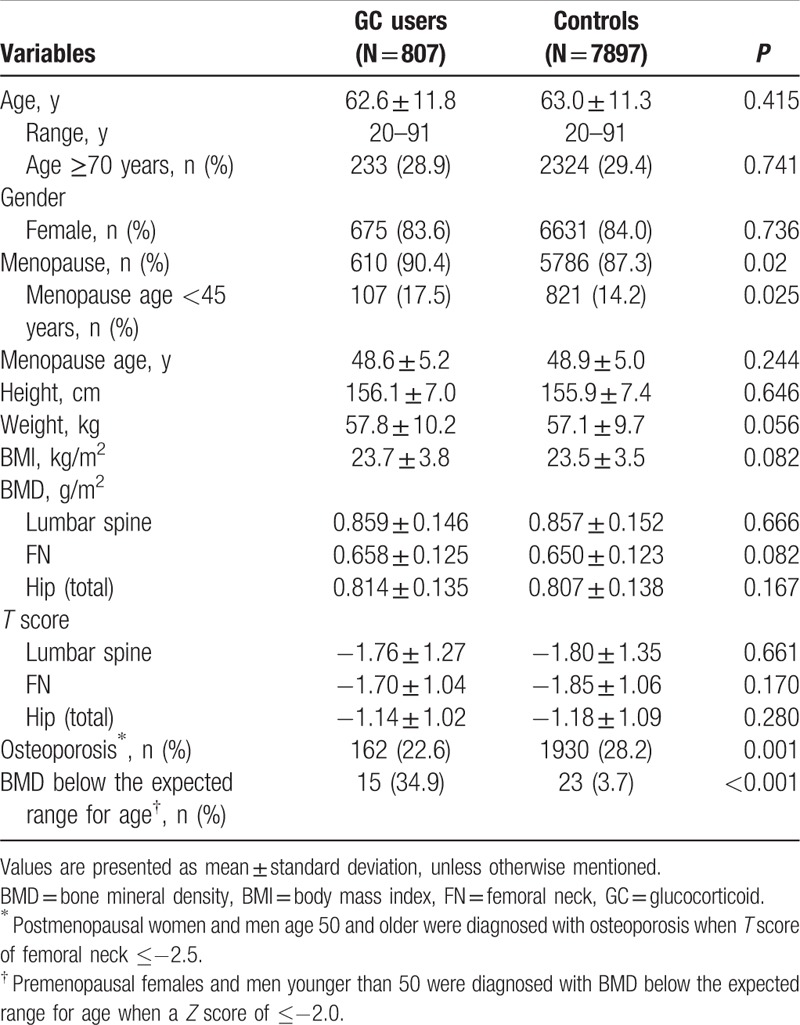
Characteristics of patients treated with glucocorticoid and sex, age-matched healthy controls.

Respective BMD values of the lumbar spine, FN, and hip (total) were 0.859 ± 0.146, 0.658 ± 0.125, and 0.814 ± 0.135 for GC users and 0.857 ± 0.152 (*P* = 0.666), 0.650 ± 0.123 (*P* = 0.082), and 0.807 ± 0.138 (*P* = 0.167) for controls (Table 1). Respective *T* score of the lumbar spine, FN, and hip (total) were −1.76 ± 1.27, −1.70 ± 1.04, and −1.14 ± 1.02 for GC users and −1.80 ± 1.35 (*P* = 0.661), −1.85 ± 1.06 (*P* = 0.170), and −1.18 ± 1.09 (*P* = 0.280) for controls. The overall prevalence of osteoporosis in postmenopause female and men age ≥ 50 years old among GC users is significantly lower than controls (22.6% vs 28.2%, *P* = 0.001; Table 1). The occurrence of BMD below the expected range for age in premenopause female and men age < 50 years among GC users is significantly higher than controls (34.9% vs 3.7%, *P* < 0.001; Table 1).

The GC users were significantly more likely to present with the following FRAX risk factors, compared to controls: previous fracture (11.9% vs 7.0%, *P* < 0.001), parental hip fracture (11.4% vs 11.2%, *P* = 0.001), RA (56.7% vs 3.4%, *P* < 0.001), and secondary osteoporosis (29.5% vs 20.1%, *P* < 0.001); however, the groups did not differ significantly in alcohol consumption (2.0% vs 1.4%, *P* = 0.220) and current smoking (5.0% vs 4.4%, *P* = 0.453; Table 2). The FRAX-determined 10-year probabilities of major and hip fractures were significantly higher for GC users than for controls (16.3 ± 11.6 vs 9.6 ± 7.8, *P* < 0.001 and 6.7 ± 8.8 vs 3.7 ± 5.4, *P* < 0.001, respectively; Table 2). The 10-year probabilities of major and hip fractures in the absence of BMD data were also significantly higher for GC users than for controls (17.9 ± 13.6 vs 9.2 ± 7.4, *P* < 0.001 and 8.9 ± 8.8 vs 3.7 ± 5.4, *P* < 0.001, respectively; Table 2). The percentages of participants at high and medium risk of major osteoporotic fracture in GC users is higher than controls (36.3% vs 9.99%, *P* < 0.001 and 32.9% vs 25.9%, *P* < 0.001, respectively; Table 2).

**Table 2:**
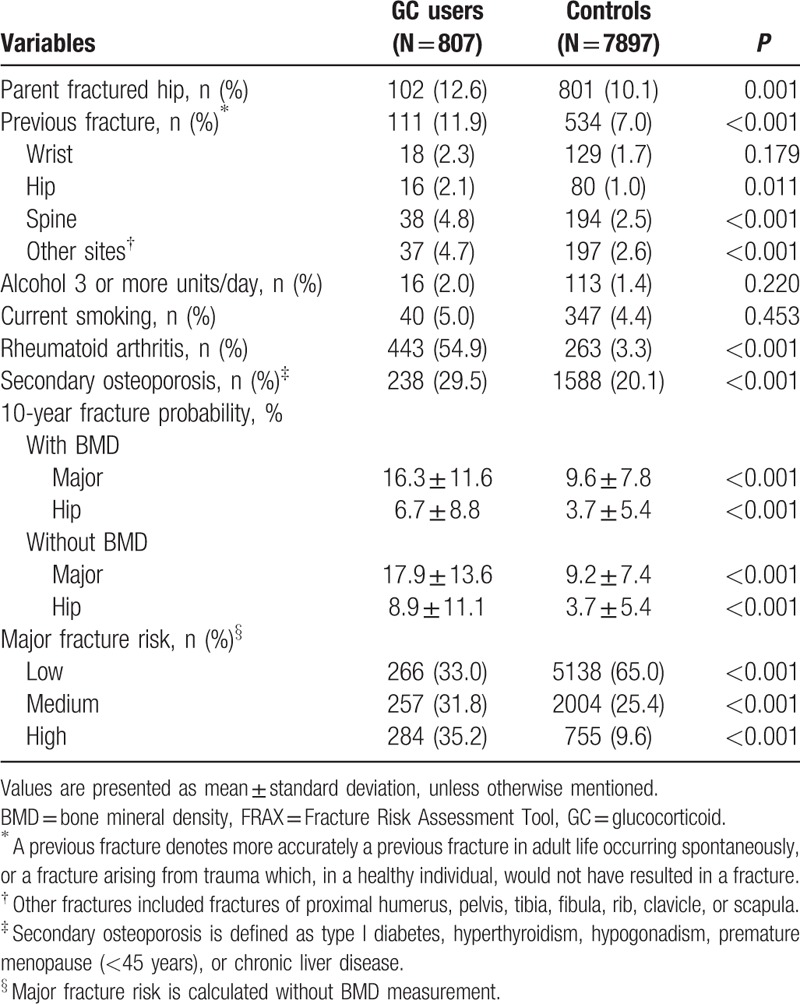
Fracture risk factors and FRAX 10-year fracture probabilities of the study participants.

The proportion of GC users with 10-year probability of major osteoporotic fracture above IIT was significantly higher than controls (for all women, 75.0% vs 10.6%, *P* < 0.001; for all men, 69.7% vs 10.9%, *P* < 0.001; Table 3). For all subjects, only 20.3% of GC users and 30.5% of controls whose fracture risk was above IIT claimed taking AOM. For all women, only 22.8% of GC users and 32.1% of controls whose fracture risk was above IIT claimed taking AOM (*P* < 0.001, Table 3). For all men, 6.5% of GC users and 22.5% of controls whose fracture risk was above IIT claimed taking AOM (*P* = 0.002). These difference are more pronounced in postmenopausal women and men age ≥ 50 years old than premenopausal women and men age < 50 years old. The frequency of self-reported current AOM usage in women with GC use were significantly higher than controls (20.4% vs 12.1%, *P* < 0.001, respectively; Table 3). There was no difference in the frequency of self-reported AOM usage between men with GC use and controls.

**Table 3:**
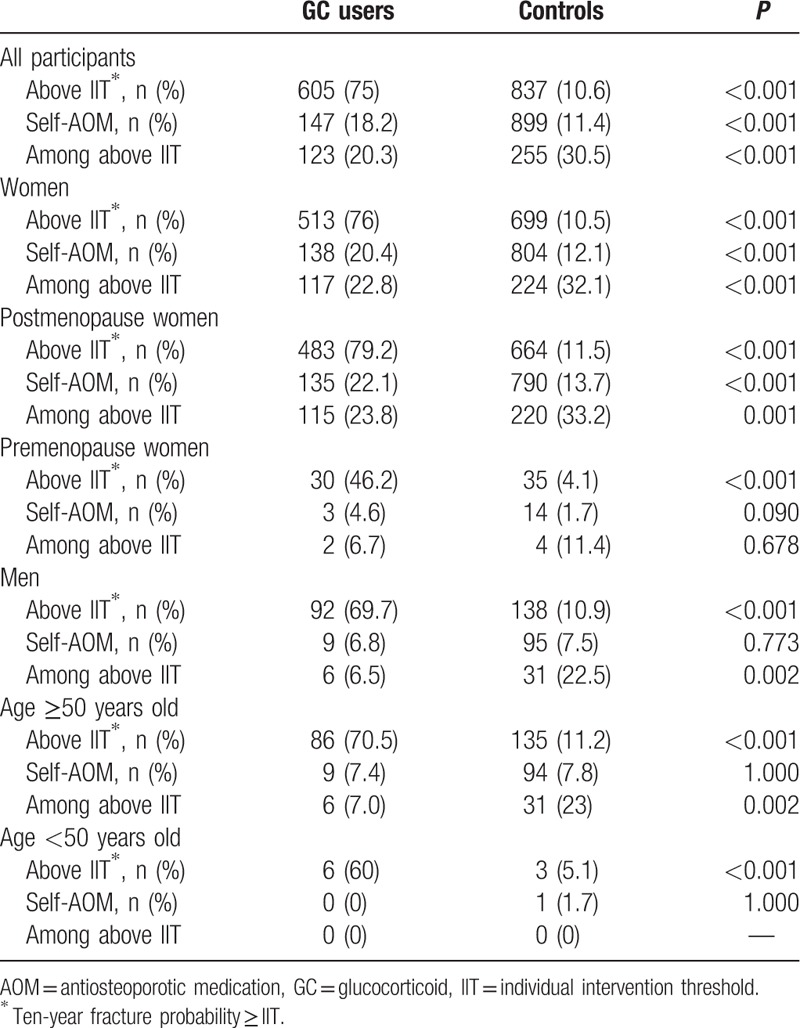
The frequency of above individual intervention threshold and self-reported antiosteoporotic medication usage.

## 4 Discussion

To the best of our knowledge, this survey-based study was the first to characterize patients receiving GC therapy and practical FRAX-based intervention implementation in a population derived from a large, nationwide Taiwan care health plan survey. Our findings revealed that approximately 4.25% (807/18,992) of adults older than 18 years were GC users. Approximately 9.1% (606/6642) of women aged 55 years and older—in other words, a population already at increased risk for osteoporotic fractures—were GC users. Our study reported a higher proportion of GC users, compared with previous published studies.^[2,3,19]^ Possible explanations for this discrepancy include the nonrandom epidemiological design of our study, the average age of our cohort (older than 60 years), and a majority of RA patients (56.7%) in GC users. A recent analysis of a national databank for rheumatic diseases in the USA reported that 38% of RA patients used GC.^[20]^ In the present study, RA patients accounted for more than half of the study group, indicating that the proportion of GC users in this nationwide screening program was relatively high.

In our study, we observed a significantly higher incidence of previous fragility fracture among GC-treated individuals relative to controls. There was no significant difference of BMD between GC users and controls. This finding agrees well with those of previous studies, in which the risks of any osteoporotic fracture and hip fracture were significantly higher among those with a history of GC use, compared to those with no history of GC use.^[8,21]^ For the same level of BMD, the risk of all fractures is substantially greater in GIOP than in postmenopausal osteoporosis.^[21]^ Thus, the risk of fractures in the context of GIOP is largely independent of BMD, likely because of an increased risk of falling, GC-induced myopathy, frailty, and changes in bone material properties that are not captured by BMD measurements.^[7,21]^ The prevalence of prior fractures, 11.7% reported in our study, is within the range of previous studies, which varies from 11% to 51%.^[21]^ It is not different, but within the range of the previous studies. A certain difference in the prevalence of fragility fractures in patients receiving GC reported in the literature might be related to discrepancies in the design of the various studies. Our study is a self-administered questionnaires survey and might have underestimated the real prevalence due to the asymptomatic nature of some fractures. Undoubtedly, CC increases the risk of fracture relative to that observed in those who are healthy.

In the present study, we examined the frequency of BMD below the expected range for age in premenopausal women and men under age 50 of both GC users and controls. The risk of fracture in premenopausal women with low BMD is much lower than that seen in postmenopausal women.^[22]^ However, as far as we know, the exact frequency of scores below the expected range for age in patients with GC has not been well described thus far. Our findings were that the frequency of expected ranges for age in patients with GC use was 7.8% and 7.8 times higher than in control subjects. There was no percentage difference of prior fracture in premenopausal women and men under age 50 of both GC users and controls. However, our study samples were relatively small. Clearly, future studies to investigate the frequency and clinical characteristics of BMD below the expected range for age in GC users are needed.

FRAX is the most widely used tool for determining 10-year fracture probabilities in individuals between the age of 40 and 90 years.^[10,23]^ The FRAX measurement only included FN BMD; however, lumbar spine, a trabecular site is very relevant as it is concerned by the effect of GCs and chronic inflammation.^[4,7]^ Thus, FRAX may underestimate the fracture probability in patients where the BMD of the lumbar spine is much lower than the BMD of FN.^[24]^ In this study, we determined that regardless of the BMD input status, significantly higher proportions of GC-treated patients had a high FRAX 10-year fracture probability and a high or medium-risk status for major osteoporotic fracture, compared with controls. These findings suggest that BMD might play a minor role with respect to GIOP prevention or treatment.

Several published guidelines for GIOP management demonstrate relatively large differences regarding the thresholds of GC dosage, BMD values, *T* score, prior fracture, or fracture probability, which are regarded as cutoff values for initiating antiosteoporotic drugs in GC-treated patients.^[11,12,24]^ None is ideal from all perspectives and there is no gold standard established by international consensus.^[25]^ Fracture probability differs markedly in different regions of the world.^[26]^ WHO suggests that each country should determine their own intervention thresholds, based on the geographical variation in fracture incidence, availability of resources, the local healthcare situation, and economic considerations. While National Osteoporosis Foundation recommends the use of fixed intervention thresholds,^[27]^ the National Osteoporosis Guideline Group in the United Kingdom encourages the use of age-specific intervention thresholds.^[28]^ In our study, we followed and modified a framework of the IOF/ECTS guideline,^[12]^ which using age-dependent FRAX probability as an intervention threshold. We observed that GC users had higher percentage of FRAX probability above IIT than controls, which indicated that higher proportion of patients commencing long-term GC therapy should be considered for AOM independently of their BMD. Establishing IIT to identify GC users for AOM might be feasible in clinical practice, as well as simple to access and use.

The IOF/ECTS provide effective guidelines for the management of GIOP. However, some issues about IOF/ECTS guideline are worthy of being discussed as follows. First, patients older than 70 years are included in a separate age group and treated directly; however, our research shows that prior fracture risk is higher among GC-treated individuals than among healthy individuals, regardless of the age group. Thus, patients older than 70 years need not be included in a separate age group. Second, the intervention threshold proposed by IOF/ECTS is calculated on the basis of a BMI of 24 kg/m^2^, but previous studies have shown that a higher or lower BMI had a marked effect on fracture probability.^[10]^ Hence, the calculation and comparison of the 10-year fracture risk for individuals from different age groups on the basis of a BMI of 24 kg/m^2^ as the standard are unreasonable. Therefore, we propose a new concept of intervention threshold, namely, the “Individual Intervention Threshold” (IIT). Decisions to recommend treatment must be based on individual assessments of risks and benefits. We delimited that intervention thresholds are “tailor-made” for each individual. BMI was set at identical BMI of subjects, not fixed at the mean of the surveyed population. For example, in Taiwan women at the age of 65 years the 10-year hip fracture probability in the absence of risk factors was 3.2% with a BMI of 20 kg/m^2^, but was fourfold lower (0.8%) at a BMI of 40 kg/m^2^. If we select a BMI fixed at 24 or 25 kg/m^2^, the influence of BMI on fracture probability might be misestimated. By using our method, one does not need to memorize the interventional threshold of any age group according to country. One only needs to use the FRAX to calculate the IIT for the first time, and then use FRAX again to calculate the 10-year probability of a major fracture for an individual. If the 10-year probability value is greater than or equal to the IIT, treatment should be administered to the individual. This method should allow clinicians to provide care or make a simple yet accurate prediction of an individual's 10-year fracture risk, and decide whether active treatment is needed.

Clinical practice guidelines have been developed to help guide physician decision-making around the treatment of GIOP to minimize bone loss and reduce fracture risk. However, only about half of the patients received recommended care as described in the guideline.^[29]^ Based on the TOPS data, treatment would be recommended in at least 70% GC users of this population based on the 2012 IOF/ECTS guideline. Self-reported AOM use in all participants (especially in men) whose fracture risk was above IIT was documented in <30%, suggesting adherence with the IOF/ECTS guideline remained low in Taiwan and indicating a possible disconnect between osteoporosis guidelines and clinical practice. Compared with controls, the proportion of self-reported AOM use in GC users whose fracture risk was above IIT is far below the proportion of individuals recommended for treatment by above IIT. This finding was not surprising, as it also confirmed what Overman's observation about a treatment gap in the management of GIOP before the guideline release.^[1]^ Despite these GIOP guideline recommendations, a systematic review noticed low levels of GIOP management. Over 80% of studies identified that <40% of chronic GC users received BMD testing or osteoporosis pharmacotherapy.^[30]^ The potential factors for low adherence of guidelines for GIOP included young age, male gender, lower glucocorticoid dose, prescription by surgery and otolaryngology specialists, and smaller clinical facilities.^[31]^ How to offer the advantage of guideline application on GC-treated patient adherence to guideline recommendations in real practice would be further investigated.

The main limitations of this study are described in the following statements. This was a self-reported population-based cohort study and was not fully verified through a medical record review; therefore, we did not know the true diagnoses of comorbidities or fragility fractures in FRAX model, and all FRAX risk factors might be inaccurate. This limitation is related to the impossibility of directly measuring factors (e.g., GC dosage, duration of GC therapy, and age of GC initiation) that might more directly lead to fracture risk in such a study. The spine fracture was ascertained based on a self-reported diagnosis, not verified by radiology reports. Thus, the influence of fracture numbers or asymptomatic fractures might have been underestimated. The use of FRAX has not been validated in individuals younger than 40 years, and therefore has not been recommended for 2.2% of the subjects in our cohort. Future research should involve an extensive study in determining the cut-off values for therapeutic intervention in GC-treated patients younger than 40 years. The TOPS study relies on self-report of current AOM use in the preceding 90 days during an in-person interview, which may cause misreporting of actual use, particularly injectable therapies that are administered at more than 90-day intervals, and likely misjudged some subjects who have mistaken “Calcium or vit-D3 supplements” as “AOM.” We were unable to provide the information of underlying chronic disease which requires GC therapies. However, a well-controlled future study may confirm the observations reported here.

The strengths of this study include the large sample size and the uniform method of data collection across study sites. Data were collected from community-based subjects in Taiwan, and few exclusion criteria were used. Physicians did not select specific patients for this study, so the overall group to whom the questionnaires were sent initially should be representative of the practices. These features helped to reduce the potential for selection and measurement biases often associated with hospital-based case–control studies. Finally, we offer a new concept of IIT as providing a simple, yet accurate rule for prediction of fracture risk and decision of treatment.

In summary, higher percentages of premature menopause, history of parental hip fracture, previous fragility fracture, RA, and secondary osteoporosis were observed in GC users relative to controls. The 10-year probability of fracture (either major or hip) was higher among GC users than controls. There is significant higher proportion of participants in GC users with 10-year probability major osteoporosis fracture above IIT than controls. These findings indicated that more GC users should receive active intervention, for example, AOM, based on above IIT, than controls. However, less than 30% of all participants (especially less than one-fourth of GC users), whose fracture risk was above IIT receiving AOM, suggesting osteoporosis is markedly undermanaged among chronic GC users in Taiwan. Clinicians who treat patients using long-term GC therapy should use IIT based on FRAX probabilities, rather than BMD, to aid treatment decisions.
